# The use of low cost compact cameras with focus stacking functionality in entomological digitization projects

**DOI:** 10.3897/zookeys.712.20505

**Published:** 2017-10-31

**Authors:** Jan E.J. Mertens, Martijn Van Roie, Jonas Merckx, Wouter Dekoninck

**Affiliations:** 1 Biodiversity Inventory for Conservation (BINCO) vzw, Walmersumstraat 44, 3380 Glabbeek, Belgium; 2 Department of Ecology, Faculty of Science, Charles University, Vinicna 7, CZ-12843 Prague, Czech Republic; 3 Department of Biology, Ecosystem Management Research Group, University of Antwerp, Universiteitsplein 1, 2610 Wilrijk, Belgium; 4 Royal Belgian Institute for Natural Sciences, Vautierstraat 29, 1000 Brussels, Belgium

**Keywords:** Focus Stacking, Compact Camera, Canon-Cognysis, Mass Digitization, Entomology, Collections

## Abstract

Digitization of specimen collections has become a key priority of many natural history museums. The camera systems built for this purpose are expensive, providing a barrier in institutes with limited funding, and therefore hampering progress. An assessment is made on whether a low cost compact camera with image stacking functionality can help expedite the digitization process in large museums or provide smaller institutes and amateur entomologists with the means to digitize their collections. Images of a professional setup were compared with the Olympus Stylus TG-4 Tough, a low-cost compact camera with internal focus stacking functions. Parameters considered include image quality, digitization speed, price, and ease-of-use. The compact camera’s image quality, although inferior to the professional setup, is exceptional considering its fourfold lower price point. Producing the image slices in the compact camera is a matter of seconds and when optimal image quality is less of a priority, the internal stacking function omits the need for dedicated stacking software altogether, further decreasing the cost and speeding up the process. In general, it is found that, aware of its limitations, this compact camera is capable of digitizing entomological collections with sufficient quality. As technology advances, more institutes and amateur entomologists will be able to easily and affordably catalogue their specimens.

## Introduction

Many museums rely on the help of volunteers for collection work (Flemons and Berents 2012; Holmes 2003). One such effort is the digitization of the vast quantities of specimens in the collections (Mathys et al. 2013; Mathys et al. 2015). Although controversy exists when describing species using photographic material exclusively (e.g., Pape (2016) and Ceriaco et al. (2016) in response), a photographic inventory of collections adds to documenting biodiversity, increases accessibility for other researchers and instances, adds to increased ecological knowledge, and helps experts and students screen specimens in an affordable way (Beaman and Cellinese 2012; Garrouste 2017).

Museums usually own a small number of digital imaging systems, constraining the digitization of collections, and can barely keep up with new additions to the collections. The professional setups typically require some level of training to use and have a high cost (€ 3.000 – € 30.000, Brecko et al. (2014)). Unsurprisingly, the price tag prevents amateur naturalists and smaller museums from acquiring such a system. Consequently, the rate of digitization not only depends on the number of volunteers but also on the infrastructure available in museums or institutes. Time-saving techniques are sometimes used, for instance whole drawer imaging (e.g., Mantle et al. (2012)). These techniques have major limitations and the resulting images often lack the resolution necessary for taxonomic accuracy or it fails to capture all required information (e.g., limited angles in which the specimen was shot or specimens covering the labels, Brecko et al. (2014); Hudson et al. (2015)).

The rapid advancement in imaging technology and software over the past few years has resulted in high-quality, user-friendly and more affordable imaging systems (e.g., the focus stacking method currently used in the Royal Belgian Institute of Natural Sciences (RBINS) and the Royal Museum for Central Africa (RMCA) of which the price is approximately € 3.000 or € 1800 when excluding the pc required for post-processing, Table 1); Brecko et al. (2014)). These systems are primarily intended to digitize type specimens, produce images for publications, retain a digital back-up of specimens prior to loans, or to avoid loans altogether. They typically involve single-lens reflex (SLR) cameras with interchangeable macroscopic (producing images on a 1:1 scale or smaller) lenses which are generally too expensive for the average volunteer to invest in. Cheaper digital cameras usually do not provide the user with the flexibility nor the image quality of an SLR camera, but manufacturers often include extra features to improve their functionality. Among these is the possibility to take macroscopic images, the quality of which has improved substantially the last decade (Pratt 2015).

**Table 1.** Comparison in price (minimum prices) and processing speed of the Canon-Cognisys setup with both TG-4’s stacking modes. ^1a^Canon EOS 600D with 60mm EF-S f/2.8 macro lens; ^1b^Canon EOS 600D with 65mm MP-E f/2.8 macro lens; ^2^off-camera flashes and platform; ^3^price for lifetime license of Helicon Focus Lite; ^4^post-processing time depends on processor type and speed among other factors; ^5^data from Brecko et al. (2014), depends on #images in stack (here: 20); ^6^already has stacking included in processing time.

	Canon-Cognisys		TG-4 manual	TG-4 internal
Camera	€ 880^1a^	€ 1500^1b^	€ 350	
Stacking set-up	€ 700		N/A	
Stacking software cost^2^	€ 100		€ 100	€ 0
Lightbox cost	€ 120^3^		€ 25	
**Total cost**	€ **1800**	€ **2420**	€ **475**	€ **375**
#images in stack	Unlimited		29	10
Image resolution	4.3 µm/pixel		1.3 µm/pixel	1.9 µm/pixel
Time to produce image	5” per image in stack		3”	13”^6^
Post-processing time^4^	17”^5^		28”	

The applicability of a compact camera was tested in view of a small digitization project of the genus *Calligrapha* (Coleoptera – Chrysomelidae) in the Royal Belgian Institute for Natural Sciences (RBINS) in September - November 2016 (Merckx et al. in prep). In this study, we assess whether a compact, (low cost) camera can replace a professional setup when it comes to digitizing entomological collections. Image quality, digitization speed, and ease-of-use were compared with the Canon-Cognisys setup and whether there are limitations to the usability of the camera.

## Methods

### Camera

The Olympus Stylus TG-4 Tough (TG-4) was used in this test. Several compact cameras focusing on macro functionality are available on the market; however, they either lack internal focus stacking (e.g., for a comparison with the Nikon Coolpix AW130, see Cameradecision (2017)) or are more expensive (e.g., some of the Panasonic Lumix line-up).

The camera is a rugged, dust- (IPX6) and waterproof (IPX8) outdoor camera with an in-camera focus-stacking feature. This camera generally gets good reviews in terms of its macro capabilities (e.g., Keller 2015). It has two stacking methods: internal stacking (in which the camera processes a stack of 10 pictures with a built-in stacking algorithm) and focus bracketing (in which the camera takes up to 30 pictures to form a stack that has to be processed by dedicated software afterwards, from here on referred to as ‘manual stacking’). In the latter, the focal step size can be set to three options: narrow, normal and wide. The differences between these settings were tested (Suppl. material 2) but since the effects were rather marginal, the narrow setting was always used. Note that the first of the 30 pictures serves as an overview and should not be included when stacking as this will lead to artefacts in the final image. The internal stacking function exports an 8MP picture, whereas the manual stacking function results in a 16MP picture. Both methods were compared to the setup currently in use at the RBINS, the Canon-Cognisys setup (for specifications, see Brecko et al. 2014). The tested compact camera is approximately four times less expensive than the Canon-Cognisys setup currently in use by the RBINS (Brecko et al. 2014, Table 1).

The camera’s capabilities were tested using five insect specimens varying in size and colour. Its image quality was compared with that of the professional setup, assessing image sharpness and level of detail and presence of stacking artefacts. In addition, distance to lens, zoom level and stacking method were altered.

### Specimen choice

Specimens from the genera *Aplagiognathus* (Coleoptera - Cerambycidae) and *Elytrimitatrix* (Coleoptera - Cerambycidae) were selected. The *Aplagiognathus* specimen was chosen for its larger size (length: 4.9 cm, width: 1.8 cm, height: 1.6 cm), uniform colour and microsculpture. The *Elytrimitatrix* specimen (length: 2.5 cm, width: 0.7 cm (2.3 cm including antennae), height: 0.5 cm) was chosen for its hairy abdomen, which often poses a problem when stacking (Brecko et al. 2014). Additionally, picture quality was assessed on images of *Polistes dominula* (Hymenoptera - Vespidae), *Forficula auricularia* (Dermaptera - Forficulidae) and *Archips podana* (Lepidoptera - Tortricidae) to test the applicability of the camera on a range of taxonomic groups.

### Lightbox and stacking software

Our own lightbox design was used, specifically made to be used with the compact camera. The body consists of a cylindrical plastic container with a hole on top that fits the lens of the camera. Inside, the top of the cylinder is lined with 59 12V, dimmable, white LED lights, covered by tracing paper to reduce light reflection on the specimens (Suppl. material 1).

Manual stacking was initially performed using the free software package CombineZP (http://alan-hadley.software.informer.com). A recent review showed that this software package underperforms in comparison with commercial packages like Helicon Focus (http://www.heliconsoft.com/heliconsoft-products/helicon-focus/) and Zerene stacker (http://zerenesystems.com/cms/home), mostly when complex structures like hairs are involved (Brecko et al. 2014). Problems with stacking (i.e., artefacts) were also encountered by us, and therefore switched to Helicon Focus as stacking software. The Helicon Focus software has a two-week free trial after which one has to pay for a lifetime license to the ‘lite’ package or the Pro package respectively, the latter adding more functions including retouching tools and batch mode, which can greatly improve the digitization workflow (e.g., stacking a large batch of images overnight).

### Tested settings

To ascertain the compact camera’s performance and to find the optimal position of the specimens, firstly the two specimens of longhorn beetles (*Aplagiognathus* and *Elytrimitatrix*) were photographed. The camera’s two stacking methods, internal and manual stacking, were visually assessed and compared to macro-photographs of these specimens from the Canon-Cognisys setup. Next, the object-lens distance (11–5 cm, with 2 cm increments) and optical zoom (1–4 times) were altered to find an optimal set of parameters. Finally, pictures of *Archips podana*, *Polistes dominula* and *Forficula auricularia* (shot in manual stacking mode with specimens at an optimal distance from the lens) were visually assessed as well, to explore applicability in a wider taxonomic range.

## Results

### Manual stacking, internal stacking, and professional setup

**Figure 1.**
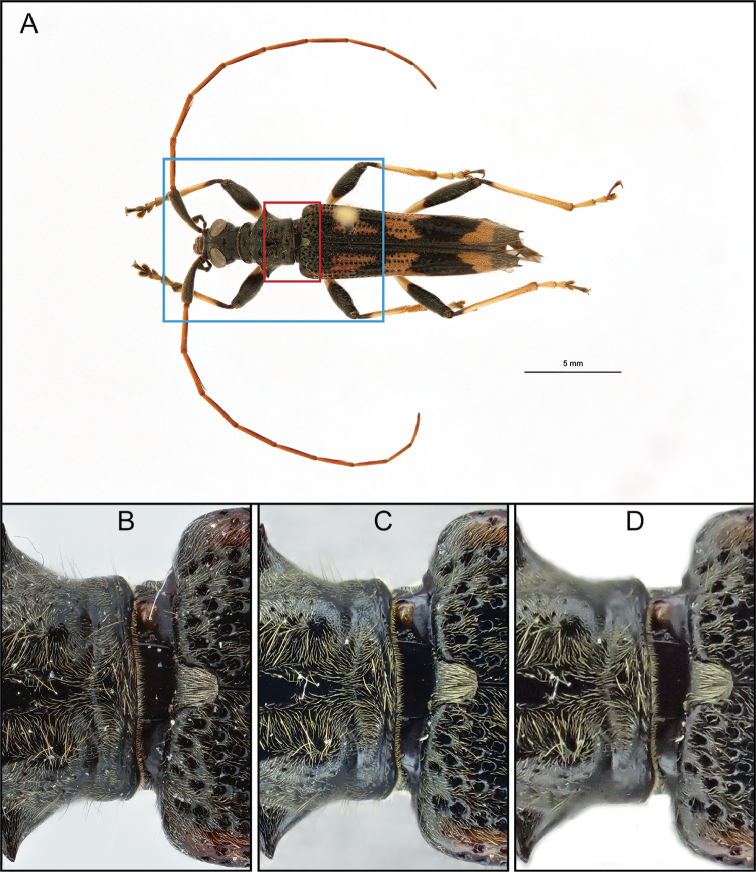
Comparison of the *Elytrimitatrix* digitized with the professional setup (**A** shot with the 60 mm macro lens and **B** with the Canon MP-E 65 mm lens), the compact camera’s manual focus stacking mode (**C**) and internal stacking mode (**D**). **A** depicts the whole specimen as would be shot for publication purposes. The red box indicates the section shown in **B, C, D** and the blue box indicates how the specimen was framed in these three images. Note that the stronger reflections in **C, D** are the result of a different lighting setup.

A comparison of the two stacking settings (internal and manual stacking) with the professional setup can be seen in Figure 1. The picture of the latter retains its sharpness towards the edges (Figure 1B), whereas images made by both stacking methods of the compact camera (Figure 1C–D) are less sharp there. Despite the softer edges of the camera’s images, the fine setae are clearly visible regardless of the stacking method. The image quality is also influenced by the positioning of the insect, the type and intensity of light and, most importantly, the stacking algorithm and software used. All three pictures conserve plenty of detail, generally sufficient for taxonomic screening. All features such as setae, elytral and prothoracic punctures, folds and dimensions can be distinguished properly, regardless of the method used. The compact camera and the professional setup are comparable in terms of usability when used for taxonomic studies.

Assuming the handling time to position the specimen is similar in all situations, the time required to finish one stacked image differs more among methods (Table 1). The Canon-Cognisys setup requires an average of 5 seconds to take one image in the stack. The compact camera we tested is faster, requiring 3 seconds to produce a complete stack of 29 pictures (ISO-100; f/2.3–4.9, depending on optical zoom, focal length: 6–18 mm) in manual mode. In both cases, off-camera image stacking is required to attain the desired result. The speed at which images are stacked strongly depends on the software package and the processing power of the computer; using the Helicon Focus software package and a Dell Latitude E5570 (i7–6820HQ Intel core processor and 8GB RAM) in all comparisons, processing a set of 29 images required on average 28 seconds. In the internal stacking mode, approx. 13 seconds are needed to make and process the final stacked picture.

### Zoom versus object-lens distance

**Figure 2.**
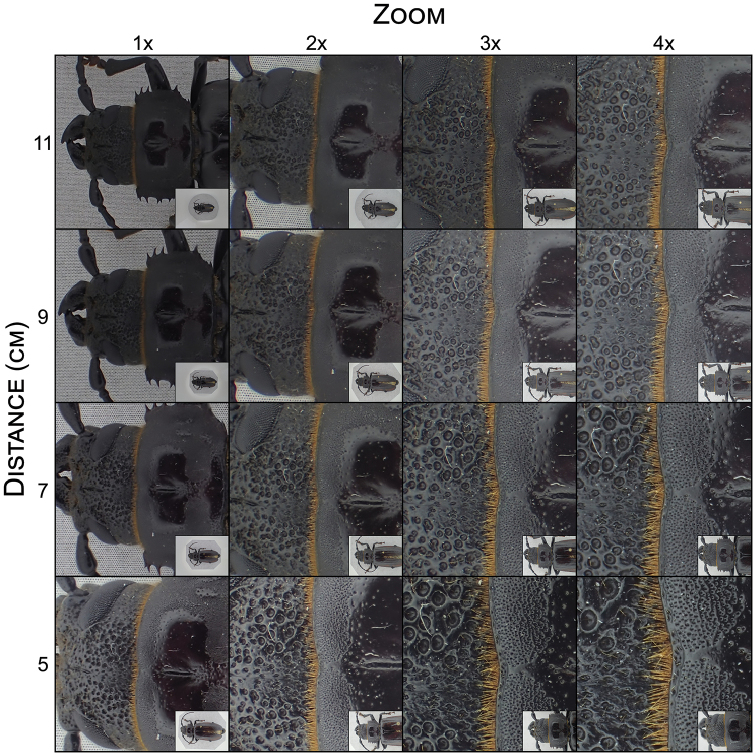
Visualization of the variation in image quality, level of detail and proportion of the specimen fitting the frame (insets) at different levels of optical magnification (1–4 times) and distance from the lens (11–5 cm). Every image, shot with the compact camera, is composed of 29 manually stacked images at the narrow setting and cropped to equal dimensions (approx. 1/24 of the original image). Quality and detail improve as lens distance decreases and/or the zoom increases at the cost of reduced depth of field and a smaller portion of the specimen fitting the image frame.

**Figure 3.**
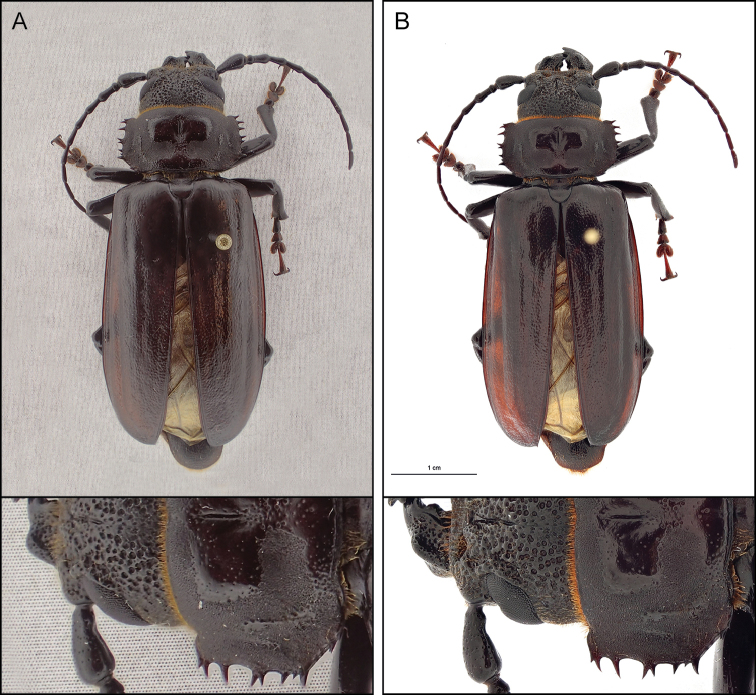
Comparison of image quality between the compact camera (**A**) and the professional setup (**B**) with the specimen occupying the same proportion of the frame. A detail is shown below. The compact camera was set up 5 cm from the specimen with the optical zoom at 1×, 29 images (narrow setting) were manually stacked. The professional setup outperforms the compact camera, producing a sharper image when specimens larger than a few centimetres are set to fill the frame optimally.

To assess any noticeable reduction in sharpness when altering the optical zoom, sample pictures at four levels of magnification were taken. No so-called ‘sweet spot’ (optimal zoom range of a lens) at a certain zoom level could be observed (Figure 2). The increased magnification does reduce the focus depth (depth of field) of the stacked image, relative to a fully zoomed-out image. This affects sharpness along the edges of the head and prothorax. However, as the specimen is placed closer to the lens, up to the minimum focus distance of 1 cm (not shown in Figure 2), the effect on the depth of field is less pronounced; the camera focuses on a point closer to the lens, but the individual distance between every image in the stack remains the same. Larger specimens, such as the *Aplagiognathus* species in Figures 2 and 3, do not fit the frame at higher zoom or closer proximity to the lens. This is where the professional setup outperforms the compact camera; producing a sharp image of the specimen as a whole and retaining more detail than a similar image shot with the compact camera (Figure 3). Moreover, the professional setup has the functionality to take images within a specific focus range, alter the step size between every image in the stack, and exchange lenses according to the specimen’s size. As a consequence, a larger range of specimen shapes and sizes can be photographed without loss of quality and resolution.

### Applicability in a wider taxonomic range

**Figure 4.**
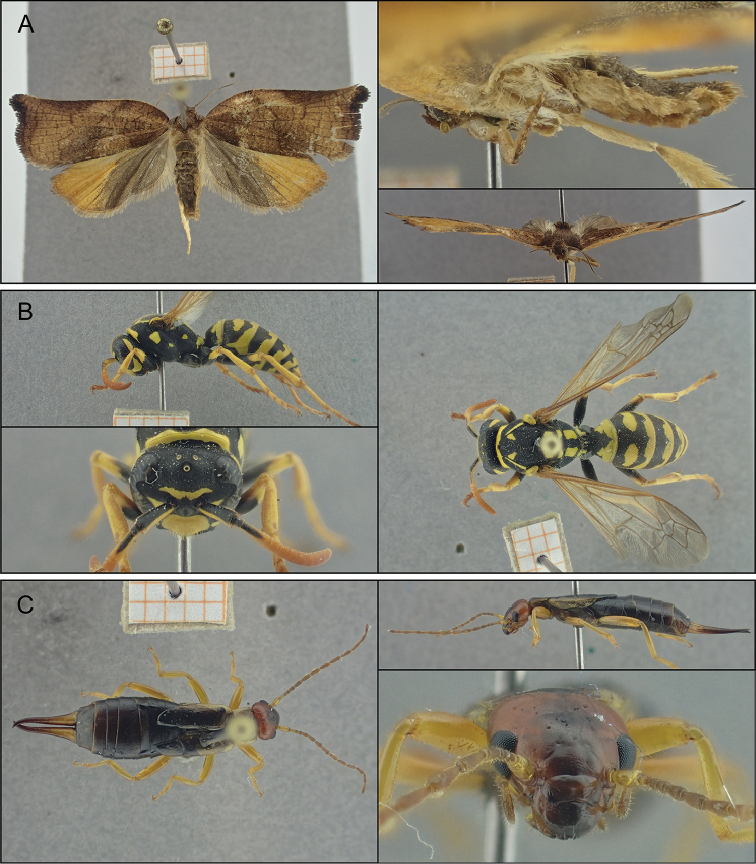
Images of different taxonomic groups, shot by the compact camera in manual mode (narrow setting). **A** large fruit-tree tortrix (*Archips podana* (Lepidoptera - Tortricidae)) **B** European paper wasp (*Polistes dominula* (Hymenoptera - Vespidae)), and **C** common earwig (*Forficula auricularia* (Dermaptera - Forficulidae)).

Figure 4 shows manually stacked images of three non-Coleopteran insects, shot by the compact camera (manual mode, narrow setting). In general, picture quality is comparable to the results shown above. The images tend to be less sharp further away from the centre, where the camera was focused. This is likely a combination of reduced corner sharpness (an optical limitation present in most lenses) and subsequent imperfect stacking of these less sharp regions of the image. Additionally, some patches of the wings in the micro-moth (Figure 4A) are less sharp than neighbouring areas. These imperfections are likely related to a combination of the relatively large distance between individual images of a stack and the limited number of pictures within a stack.

## Discussion

### Internal stacking versus manual stacking

The internal stacking and the manual stacking mode of a compact camera were compared with a professional museum imaging setup. We found that in terms of picture detail and centre sharpness, the compact camera’s images are often comparable to the professional setup when it comes to image quality. However, pictures shot with the first, likely due to its limiting 10 (internal stacking mode) or 29 (manual stacking mode) images per stack and limited options defining the focal distance between each image (“wide”, “normal” and “narrow”), were more prone to local loss of focus (e.g., along the edges). The latter is especially clear when the object of interest spans the whole frame. The narrow setting results in marginally sharper images, barely noticeable in areas with more depth. However, due to the limited focus range, extremities (i.e., legs and antennae), and ‘deeper’ parts of the body fall out of focus. This can be alleviated by focusing exactly in the middle (i.e., mid-depth) of the specimen, for example more towards the head instead of the highest point of the abdomen. The normal setting usually solves this problem, broadening the focus range sufficiently to include the whole specimen. The professional setup is more versatile as its number of images in a stack can be adjusted, based on a predefined focus range and step size. Decreasing the step size results in a smoother transition from slice to slice and setting the focus range ensures the fore- and background to be out of focus. Therefore, the professional setup can provide a sharp image across the whole specimen, regardless of its shape or size.

The relatively small sensor size of the TG-4 (6.17 mm × 4.55 mm), when compared to any SLR camera (e.g., Canon APS-C: 22.3 mm × 14.9 mm), is unable to capture the amount of detail the professional setup can and, together with the limited number of images in a stack, can result in a less detailed image with parts of the frame being less sharp, especially when framing a large specimen (i.e., fully zoomed out and more distant from the lens). Nevertheless, the images shot by the compact camera retain key taxonomic features such as hairs and punctures. Additionally, the abovementioned stacking imperfections are often corrigible in the stacking software. This, however, requires the user to select manually which parts of one slice should be used in the final stacked image, increasing the processing time per stacked image.

When comparing the internal and manual stacking, it was found that a sharper image is achieved in manual stacking mode. This result is influenced by several factors, including the higher number of pictures in a manual stack (29 versus 10 in internal mode), the higher image resolution to 16MP (instead of 8MP) and the possibility to adjust focus range from narrow to wide. We should note, however, that the quality of manually stacked pictures also depends on the capabilities and limitations of the stacking software. Results varied when stacking the same batch of images in the freely available CombineZP software after which we opted to use the professional Helicon Focus software. Even though manual stacking is more time consuming (28 seconds per stack versus 13 seconds with internal stacking), most of this work can easily be batched in the stacking software and ran without user interaction using the Helicon pro license. The time spent transferring, organising, and labelling files onto the computer to prepare for Helicon’s stacking depends on the number of images and can easily add several minutes to the process. Another advantage of the stacking software, are the options to fine-tune several stacking algorithm parameters like smoothing and radius (http://www.heliconsoft.com/helicon-focus-main-parameters/) to improve the final image quality.

Apart from technical aspects, internally stacked pictures can easily be checked for incorrect focus on the camera’s LCD screen whereas errors in manually stacked pictures due to some parts of the specimen being not in focus are usually only discovered after processing. We would only recommend the internal stacking mode when no workstation and/or sufficient hard drive space are available (i.e., 29 image slices of one specimen can take up to 100Mb unstacked and 5Mb when stacked, whereas an automatically stacked image usually takes up below 2Mb).

### Zoom versus object-lens distance

The optical zoom did not substantially affect image quality. The feature that mattered most was the distance to the lens; the smaller the distance between the specimen and the lens, the more details could be discerned (e.g., punctuation, hairs). Nevertheless, there is a subtle functional difference between zoom versus distance to lens. Increasing the optical zoom slightly compresses the image stack, resulting in a smaller focus range. Zooming in is therefore practical when capturing details (e.g., microstructures and small setae) but less so when framing a specimen that requires more focus depth (e.g., a frontal view or legs stretching down far below the specimen’s body). Consequently, it is recommended to position such specimens closer to the lens instead of zooming in to profit from the larger focus range, the opposite is true for small specimens. Even though the focus compression effect is small, it is easy to take into account when positioning the specimen and might help retain more details in the stacked image. It could also prove to be helpful to adjust the focal step size, where a narrow step size often generates marginally better results, but could miss some parts of bigger specimens whereas a normal or wide setting wouldn’t.

One other drawback of using the compact camera tested in this study on larger specimens is the trade-off between detail and a full view of the specimen (Figure 3). Taking images of large specimens with the highest possible quality (in terms of detail) is impossible unless several pictures, taken by moving the camera above the object, can be ‘stitched’ together (using so-called micro panorama software). This would require more processing time and could again decrease the overall image quality due to misalignments. In practice, however, we found that this procedure is unfeasible; at such close distances, parallax differences cause large shifts of objects closer to the lens compared to more distant ones, making it impossible for the program to stitch images together. In this respect, we conclude that the setup is perfect for small specimens with a maximum of around 1–2 cm in length, but leads to decreased quality for bigger specimens because of the greater distance from the lens required to fit the specimen in the frame.

### Taxonomic range

In light of applicability to a wider taxonomic range than just Coleoptera, we tested the manual stacking mode to three other specimens: a European paper wasp (*Polistes dominula* (Hymenoptera - Vespidae)), a common earwig (*Forficula auricularia* (Dermaptera - Forficulidae)) and a large fruit-tree tortrix (*Archips podana* (Lepidoptera - Tortricidae)). In general, the resulting pictures are of good quality and detail. Some errors can remain, however, due to the limited focal step size adjustability, for example a slight tilt of the wing in Lepidoptera can cause certain parts to be out of focus. For large specimens or large-winged insects, this might pose an inconvenience. Note that these specimens surpassed the ‘ideal’ range of 1–2 cm. Additionally, the typical reduction of image sharpness towards the corners might influence the optimal positioning of a specific specimen. We recommend to always evaluate this beforehand.

## Conclusions

When it comes to digitization of entomological collections, it seems that compact camera models such as the TG-4, used in this study, cannot out-compete professional imaging systems such as the Canon-Cognisys setup. This is in part due to the limited number of images in a stack and lower versatility when it comes to specimen dimensions. In situations where higher quality images are preferred (e.g., type material), specimens should be digitized with a professional, high quality setup. Nevertheless, compact camera models are a valuable addition to the professional setup for rapid specimen digitization. The ease of use and affordability could help reduce the digitization backlog of large museums or be the primary means to digitize specimens of personal collections or smaller institutes. This camera performs best for small specimens (around 1–2 cm) because they can be positioned closer to the lens without falling out of frame or reach the camera’s minimum focus distance. The manual stacking function, with 29 images, generates the best results, but has a significantly longer (post-)processing time. The latter can however be avoided by investing in a professional stacking software package with batching functionality. We do not recommend using the automatic stacking mode unless no workstation with stacking software or sufficient hard drive space is available. It generates a lower quality image; however, depending on the taxonomic group, it should still show key taxonomic features with sufficient detail to be useful to experts.

Trade-offs aside, budget compact cameras are constantly improved upon, including their macro capabilities and functions. The emergence of focus stacking features is an important step towards affordable professional-grade macroscopic images. Consequently, digitization of insect specimens has become affordable for most people and institutes. The internal stacking function could eliminate the cost of a dedicated stacking program and further costs (i.e., lightbox) are negligible. Together with a good volunteer program, a combination of a professional setup for type specimen digitization and compact cameras with focus stacking functionality could drastically speed up digitization efforts in an affordable way.

## Supplementary materials

Supplementary material 1 Figure S1 Data type: PNG File (.png) Explanation note: Lightbox setup, the camera rests on top of the cut-off bucket, its lens protruding though the hole in the middle. The specimen is usually shielded from direct light by a free-standing cylinder of tracing paper (not depicted). The LED strips on the inside are powered through a 12V adapter. This dataset is made available under the Open Database License (http://opendatacommons.org/licenses/odbl/1.0/). The Open Database License (ODbL) is a license agreement intended to allow users to freely share, modify, and use this Dataset while maintaining this same freedom for others, provided that the original source and author(s) are credited. Jan E.J. Mertens, Martijn Van Roie, Jonas Merckx, Wouter Dekoninck

Supplementary material 2 Figure S2 Data type: JPG File (.jpg) Explanation note: Comparison of the narrow (**C, F**), normal (**A, B, D, G**) and wide (**E, H**) focal step size in two specimens of different ‘depth’ (**A, C–E**: *Allochroma* sp., 2 mm deep; **B, E–H**: *Doryphora* sp., 12 mm deep, measured from top of elytra to lowest tarsi). The narrow setting is marginally sharper in some areas; however, deeper parts of the specimen are not in focus. The wide setting produces artefacts around some of the edges, sometimes resulting in less sharp regions.
 This dataset is made available under the Open Database License (http://opendatacommons.org/licenses/odbl/1.0/). The Open Database License (ODbL) is a license agreement intended to allow users to freely share, modify, and use this Dataset while maintaining this same freedom for others, provided that the original source and author(s) are credited. Jan E.J. Mertens, Martijn Van Roie, Jonas Merckx, Wouter Dekoninck
