# Supplementary figures and images for: The use of low cost compact cameras with focus stacking functionality in entomological digitization projects
Source: Zookeys. 2017 Oct 31;(712):141–54. doi: 10.3897/zookeys.712.20505 (PMC5674212; doi:10.3897/zookeys.712.20505)

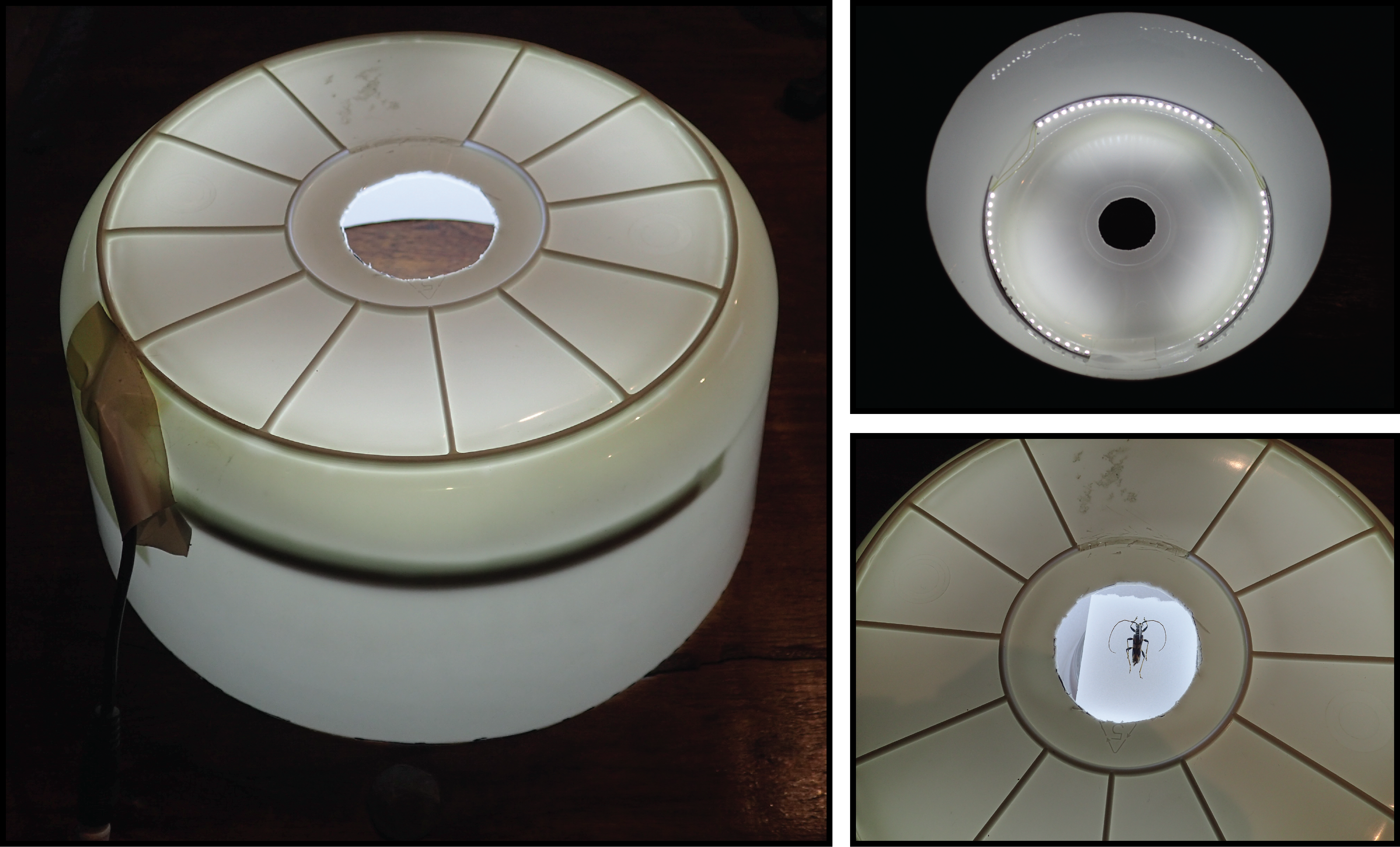

Supplement: Supplementary material 1 — Figure S1 [file zookeys-712-141-s001.png]

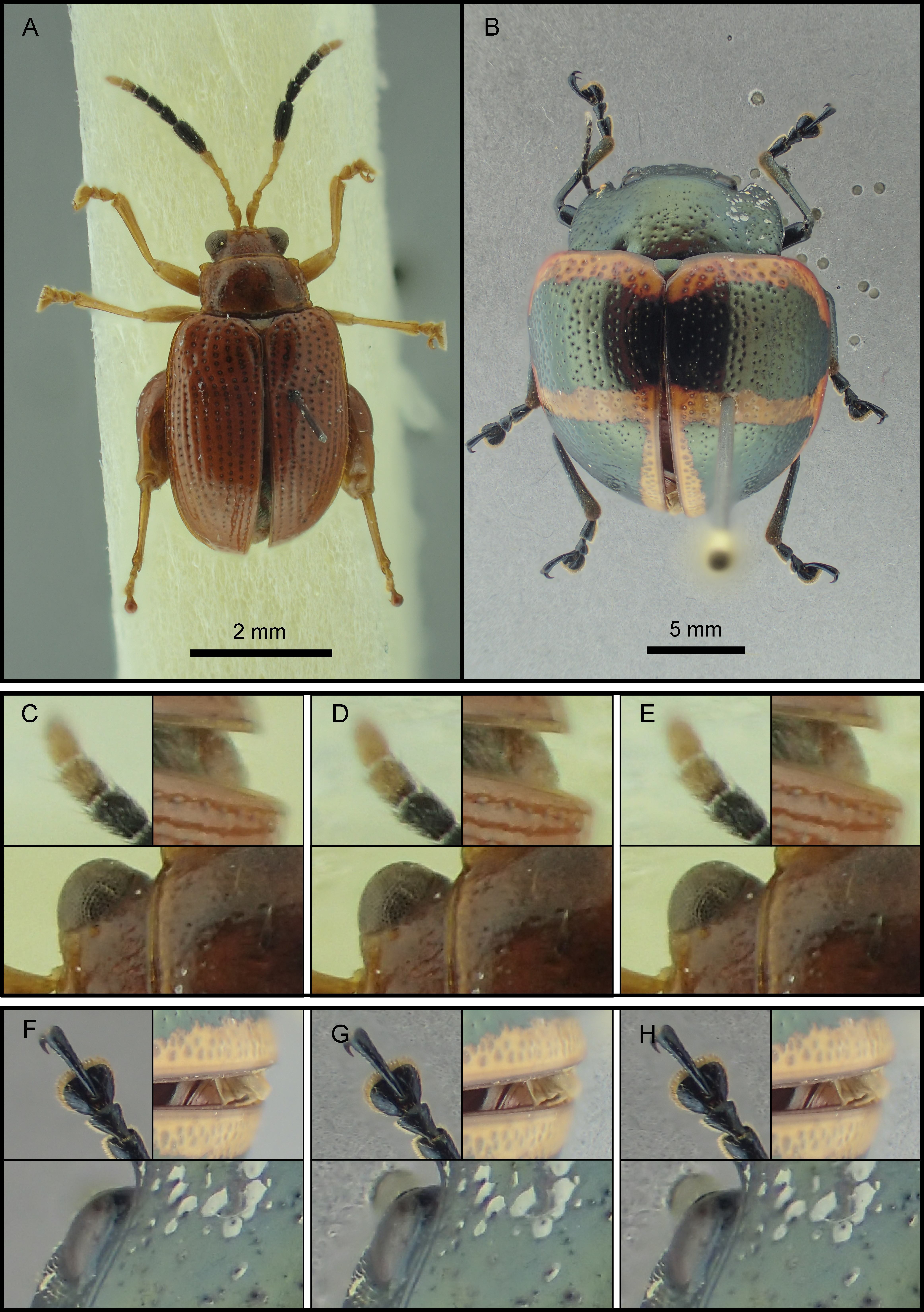

Supplement: Supplementary material 2 — Figure S2 [file zookeys-712-141-s002.jpg]
